# Heart Rate Behavior in Speed Climbing

**DOI:** 10.3389/fpsyg.2020.01364

**Published:** 2020-07-07

**Authors:** Franz Konstantin Fuss, Adin Ming Tan, Stefanie Pichler, Günther Niegl, Yehuda Weizman

**Affiliations:** ^1^Smart Products Engineering Program, Swinburne University of Technology, Melbourne, VIC, Australia; ^2^Institute of Sports Science, University of Vienna, Vienna, Austria; ^3^Climb-on-Marswiese, Sportstättenverein Marswiese, Vienna, Austria

**Keywords:** speed climbing, heart rate, psychological pressure, anaerobic, acceleration, rules of climbing

## Abstract

Speed climbing is an Olympic discipline within the combined sport climbing event in 2020 for the first time. Speed climbing is a high-speed and anaerobic exercise against gravity over a few seconds with extreme psychological pressure. Although there is some literature on heart rate (HR) when lead climbing, there is no literature on the behavior of the HR when speed climbing. The HR of seven near-elite participants was measured with a Polar HR monitor while climbing a 10- and 15-m wall, respectively, three times each, with pauses of 5 min between the first and last three climbs and a 20-min pause between the third and fourth climb. The average climbing times on the 10- and 15-m walls were 9.16 ± 3.06 s and 14.95 ± 3.14 s, respectively (data pooled between climbing heights). The peak HR on the 10- and 15-m walls were 164.57 ± 7.45 bpm and 176.43 ± 8.09 bpm. The rates of change in HR were as follows: average HR acceleration before peak HR, 2.53 ± 0.80 bpm/s; peak HR acceleration before peak HR, 4.16 ± 1.08 bpm/s; and average HR deceleration after peak HR, −0.98 ± 0.30 bpm/s. The average HR during the pauses ranged from 105.80 to 117.89 bpm. From the results, in comparison to the literature, we conclude that athletes, trained for sustaining high physical exertion and psychological pressure, have a far smaller HR acceleration than untrained people during light and unstressful exercises. Furthermore, the current rule that athletes shall have a minimum resting time of 5 min between climbing attempts during a speed climbing competition seems justified as sufficient time for HR recovery.

## Introduction

Speed climbing is one of the three disciplines of combined sport climbing, an Olympic discipline in 2020 for the first time. Speed climbing is a unique sports discipline that requires high-speed, high-power, and precise non-cyclic movements with a full-body workout (all four limbs), by lifting the body center of mass by ∼13 m (on the 15-m wall) against gravity at maximum possible speed, concentration, and extreme psychological pressure. These are conducted over a very short period of time, specifically only over a couple of seconds, and with a very high risk of failure. The current world records (as of the submission of this paper) are 5.48 s for men and 7.10 s for women (International Federation of Sports Climbing [IFSC], 2019a), corresponding to an average climbing speed of 2.74 and 2.11 m/s, respectively.

There is no other Olympic discipline comparable to the intensity of speed climbing. Other highly anaerobic disciplines include the following:

– Running, 100-m sprint. It has the same cyclic movements of the legs over the entire distance (starting excepted) and low failure risk as compared to climbing. In addition, the cumulative vertical upward displacement of the body center of mass (COM) is small compared to speed climbing [Usain Bolt: 41 steps over 100 m (Maćkała and Antti, 2013), average and maximal vertical displacement of the COM per stride equals 45 and 49 mm, respectively (Čoh et al., 2018), resulting in a total upward displacement of 1.85–2 m over 100 m];
– Speed skating, 500-m sprint. Same as running, with zero vertical upward displacement of the COM;
– Cycling, flying 200-m time trial. Same as running, with minimal failure risk and zero vertical upward displacement of the COM;
– Wheelchair racing, 100-m sprint. Same as cycling, but using the arms instead of the legs;
– Swimming, 50-m freestyle. Same as cycling, but with zero failure risk, and with movements of all four limbs.

There is some literature on how the heart rate (HR) behaves in short anaerobic actions, mostly shown by means of HR profiles, i.e., plotting the HR against time.

For example, Svensson (2007) investigated the HR behavior in blocks *“consisting of 5 running cycles of short (2 m* × *15 m) and long (50 m) high-speed runs with a 90-s rest period in between blocks.*” However, the actual running time is not shown with respect to the HR profile.

Bogdanis (1994) investigated the HR in sprint cycling and compared the “peak heart rates” of the different tests. However, the “peak heart rates” were measured in general only every 30 or 60 s, with one datum at the end of a sprint. This method can be deceptive and be misinterpreted in the sense that the peak HR always occurs at the end of the exercise, with a subsequent immediate decrease.

This is not the case as shown by the following:

– Weinstein et al. (1998) in the 30-s Wingate Anaerobic Test on a cycle ergometer, where the peak HR occurred within 5 s after the end of the test.
– Casuso et al. (2014) in swimming, “*5 repetitions of maximal 100 m swimming bouts separated by 5 min of recovery*,” where the “*HR peak was located during the last 10 s of the sprint and the first 10 s of the recovery.*”

**FIGURE 1:**
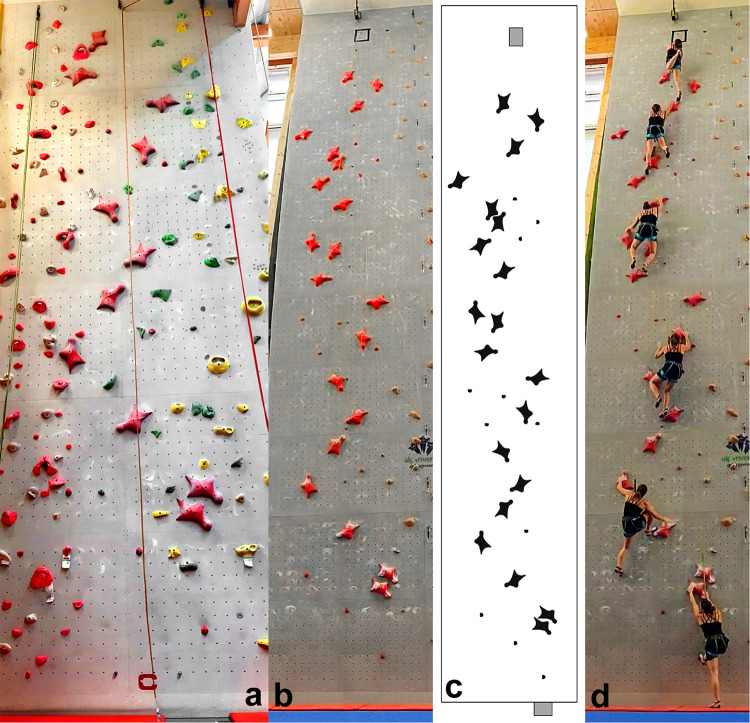
Climbing walls used in this study; **(a)** 10-m walls; **(b)** 15-m walls; **(c)** hand- and foothold pattern of the 15-m wall according to the IFSC (International Federation of Sports Climbing [IFSC], 2014), and **(d)** participant climbing the 15-m wall.

Sandvei et al. (2012) investigated the HR behavior in “*Sprint interval training consisting of 30 s sprints*…*with a 3 min rest between each sprint.*” The authors did not indicate the sprint time with respect to the HR profile. However, from Figure 1 in their paper (Sandvei et al., 2012), it became clear that the HR peaked ∼30 s after the end of the sprint, thereby still increasing over the first half minute of the rest period. Subsequently, the HR decreased over the next 2 min, leaving ∼30 s for preparing for the next sprint (in the test persons of Sandvei et al., 2012). This pattern of HR behavior raises the question whether a 3-min rest between the sprints suffices for reducing the HR to a steady state, or whether the HR would have dropped further if it was not for the preparation phase for the next sprint.

Considering this issue for speed climbing, according to the IFSC Rules (International Federation of Sports Climbing [IFSC], 2019b), specifically Rule 9.14, “…*competitors shall be afforded a minimum resting time of five (5) minutes between attempts on the route(s).*” Are these 5 min selected adequately, if not based on scientific proof, for allowing the HR to reach a steady state, i.e., a relatively constant HR over an acceptable amount of time?

To the best of the authors’ knowledge, there is no literature source on the behavior of the HR during speed climbing.

However, there is some general literature on the HR in climbing. In fact, even one HR profile while lead climbing was published (Figure 4.4 of Giles, 2017), where the HR seemed to decrease immediately after completing the climb. The behavior of the HR in lead climbing is covered in detail by literature review paper such as Sheel (2004) and by two recent reviews: Michael et al. (2019) and Saul et al. (2019). As such, only the most important aspects of the HR in lead climbing are summarized subsequently.

The increase in the HR in climbing is multifactorial and depends on the following factors:

(1) The *difficulty of the route—*the more difficult the route, the higher the HR (Sheel et al., 2003). The more inclined an overhanging wall is, the more difficult the climbing route is (Fuss and Niegl, 2008b), which affects the HR (Baláš et al., 2014).
(2) Repetitive *isometric contractions* of the forearm musculature impede the local blood flow and lead to a higher heart rate and blood pressure (Astrand et al., 2003).
(3) While climbing, the *arms* are held *in the overhead position* for most of the time, which increases the HR (Mermier et al., 1997).
(4) *Psychological stress and anxiety* produce a higher HR (Mermier et al., 1997; Booth et al., 1999; Sheel, 2004; Draper et al., 2008).
(5) The more *experienced* rock climbers are, the lower the heart rate is (Sheel, 2004; Baláš et al., 2014).
(6) *Outdoor climbing* evoked a higher heart rate response than indoor climbing (Booth et al., 1999).

The aims of this research result from the knowledge gap identified above:

(1) How does the HR behave while speed climbing, and how does the HR profile look like, when climbing the same route several times, separated by a 5-min resting period?
(2) Are 5-min resting periods sufficient for recovery of the HR before the next climb?

## Methods

### Rationale of the Method

This study was conducted *during* the training of speed climbing, and the HR was measured *during* speed climbing bouts and pauses (intervals) between the bouts. Point to note is that this study is not related to research into *interval training programs*, let alone *HR-based interval training programs*. The reason for this approach is twofold:

(a) The initial aim of this study was to conduct the collection of data during a speed climbing competition. Although we would have had access to many speed climbers in the same place and at the same time, the intended approach was not advisable, if not impossible, for various reasons. Instrumenting the participants with wearable devices would have severely disrupted the competition. Several ECG-based chest belts (Polar H7) were required, and it takes ∼10 min for preparing one climber by putting the chest belt on, explaining the procedure, and having the consent form filled in. Neither the judges of the speed climbing competition nor most of the participants would have approved wearing the chest belt as a certain tension is required for maintaining a good contact between the ECG electrodes and the skin. This belt tension could have distracted the climbers if they are not used to wearing such a belt. From own experience, climbers are very sensitive to distractions during competitions and become highly emotional when failing (falling off the wall) because of a distraction. It would have been probably easier to conduct HR measurements during competitions with optical HR sensors incorporated in smartwatches. However, it is well established that these optical sensors grossly underestimate the HR (Duking et al., 2016). Thus, ECG-based chest belts are the preferred option for collecting accurate data. As such, the study had to be carried out during speed climbing training. One could argue that the level of arousal is different under training and competition conditions, but investigating this under competition conditions had been ruled out as stated earlier. Two of the authors of this paper have experienced these problems before, namely, longer disruptions of a competition because of incorrect placement of an instrumented handhold (Fuss and Niegl, 2008a).
(b) The interval lengths (pauses between climbing bouts) were selected based on the international rules of speed climbing (IFSC, International Federation of Sports Climbing [IFSC], 2014), namely that the rest periods between two climbs must be at least 5 min. The objective of applying these rest periods was to verify that the HR decreases during these 5 min and by how much it actually decreases. During competitions, not all the participants have to go through two or more climbing ascents and therefore are not exposed to at least 5-min pauses. A properly designed study, carried out during a training process in contrast to during a competition, with a predefined number of pauses related to climbing ascents on 10- and 15-m wall, will provide more consistent numbers of datasets across the participants. From a physiological point of view, the minimum pause of 5 min seems to be correctly selected, as speed climbing is a short (5–15 s) “all-out” full-body exertion, whose primary energy system in use is the adenosine triphosphate–phosphocreatine system (ATP-PCr; Foss and Keteyian, 1998; Wilmore and Costill, 1999; Conley, 2000; McArdle et al., 2001). Periods of 3–5 min are necessary for the complete recovery and replenishment of the ATP-PCr energy system (MacDougall and Sale, 2014; Carmer et al., 2015). Conley (2000) suggested that work-to-rest ratios of 1:12–1:20 should be applied to 5–10-s long high-intensity exercises. Lloyd Jones et al. (2019) applied work-to-rest ratios of 1:8, 1:10, and 1:12 to 6-s exercise bouts of high-speed cycling. Along these lines, applying a work-to-rest ratio of 1:12 to a speed climbing bout of 15 s results in a 3-min pause required for replenishing the ATP-PCr energy system. A 5-min pause, prescribed by the rules, seems therefore appropriate. However, it is unknown how the HR behaves over these 5-min pauses. At least, it can be expected that the HR increases further immediately upon completion of the speed climb. At this point, the excess postexercise oxygen consumption (EPOC; Carmer et al., 2015) is established and highly engaged during the 5-min recovery. This increased rate of oxygen intake, along with perceived psychological stress, will continue to drive up heart rate after the climb. As such, both psychological overload and physiological stress will cause a rise in HR immediately after “all out” exertion. In this context, the behavior of the HR immediately after the climb and at the beginning of the pauses was of particular interest in this study.

In this study, recruiting the number of participants was a challenge: (1) participants have to agree to participation (consent according to Research Ethics); (2) in contrast to lead climbing and bouldering, the number of speed climbers is far less, as speed climbing is athletically very demanding and is not suited for casual climbers (whereas for the other two climbing disciplines, most active climbers are casual ones); (3) a climbing gym is required that has both speed walls at their disposal (10-m wall, and the international 15-m wall, considering that 15-m walls are very rare); (4) the participants must be preferably members of a national or regional team and participating in national or regional competitions, to guarantee at least a subelite level; and (5) all the participating speed climbers should be organized by their local climbing gym, and preferably by their coach, so that the HR data can be collected during their standard training process (and not having an experiment staged which could have a different psychological effect than their standard training process). Furthermore, working with each climber required a time commitment of 70 min in total (including preparation and debriefing).

The number of participants could have been solved when measuring participants of a speed climbing competition; however, this was already ruled out based on other concerns, as outlined above.

Despite having only seven participants, we see this preliminary study as a starting point for further research into speed climbing, specifically for multicenter trials to achieve a higher number of participants, with a tested and established method—outlined subsequently.

### Speed Climbing Route

The climbing routes used in this study were a 10-m wall (top-roping with belayer; Figure 1a) and a 15-m wall (top-roping with automatic rope brake; Figures 1b–d). Both routes complied with the international rules, composed of one specific hold type with the standard handhold pattern as specified by the IFSC (International Federation of Sports Climbing [IFSC], 2014; Figure 1c). The reason why both 10- and 15-m speed climbing routes were used was to investigate the difference in reduction in HR after a 10- and a 15-m climb.

### Participants

The HR of seven climbers was measured [5 female and 2 male climbers; 19.7 ± 2.1 years; speed climbing experience, 2.38 ± 3.45 years; best 15 m speed climbing time, 12.88 ± 4.07 s; body height, 1.68 ± 0.05 m; body mass, 61.4 ± 10.8 kg; body mass index (BMI), 21.55 ± 2.69 kg/m^2^].

This study was granted ethics approval by the Swinburne University Human Ethics Committee (approval no. 20191290-1680) and adhered to the Declaration of Helsinki.

### Experimental Procedure

The HR data were measured during the speed climbers’ standard training process with a chest-worn Polar H7 chest belt, which is an ECG-based heart rate monitor (Polar, Kempele, Finland). The chest belt was placed directly below the sternal part of the pectoralis major muscle (as per recommendations of the manufacturer; Polar, 2020), centered around the xiphoid process. The data from the chest belt were transmitted to a Polar A300 receiver, which is usually worn on the wrist but was attached to the climbers’ harness at the waist level in this study. The HR data were recorded at a sampling rate of 1 Hz. After each climb, the data were downloaded from the Polar A300 to the laptop via Polar Flow web service.

The Polar system and a stopwatch were switched on simultaneously ∼3 min before the first climb and switched off 10 min after the last climb. After a warm-up session, the participants had to climb the 10-m wall three times, with 5-min pauses in between, followed by a 20-min rest interval. Subsequently, the participants climbed the 15-m wall three times, equally with 5 min pauses in between. The stopwatch served for recording the split times at the beginning and end of each climb, in order to synchronize the climbing actions with the HR data. The accuracy of the data synchronization process was verified in the lab over 40 min, by switching on the Polar system and the stopwatch simultaneously, and at every full minute, the Polar sensor was put on the chest for 5 s.

Measurements of the HR with a Polar heart rate monitor (e.g., H7) are common in climbing, used, e.g., by Baláš et al. (2014) and Giles (2017). The Polar H7 was validated by Giles and Draper (2017), against three-lead ECG as a gold standard for correction methods of RR intervals. Gaynor et al. (2019) validated Fitbit Charge HR, Polar H7 heart rate sensor, and Masimo SET Rad-5v against a three-lead ECG during continuous and interval exercise. The Polar H7 heart rate sensor exhibited the highest accuracy with the ECG, with a bias of 0 ± 1 bpm (Bland–Altman method) during both exercises. Unsurprisingly, the H7 Polar belt was used as a gold standard in several studies, such as by Hernando et al. (2018), who validated the Apple Watch against the Polar H7; and by Schubert et al. (2018), who validated an optical heart rate sensor (Polar^®^ OH1) against the Polar H7.

### Data Processing

**FIGURE 2:**
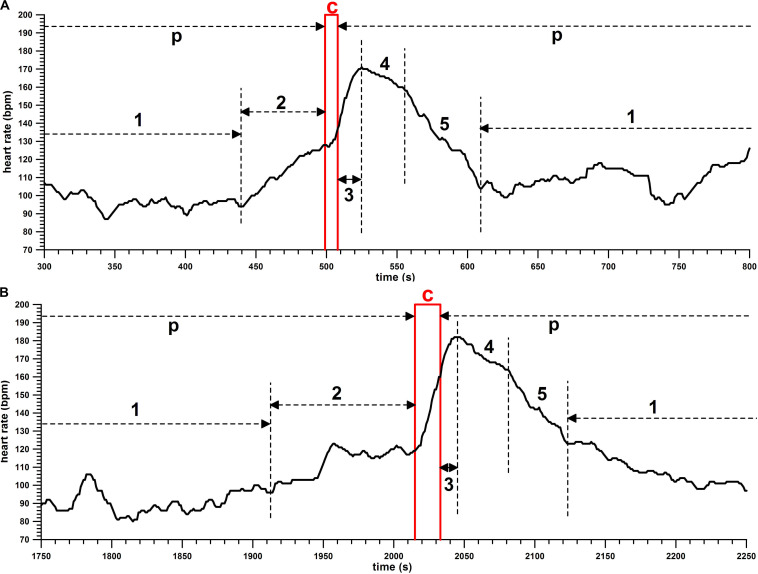
Heart rate (HR; bpm) against time (s), samples of HR profiles of **(A)** 10-m and **(B)** 15-m climbs; p, pause; c, climb; 1, rest with low HR; 2, prestart activation immediately before the climb; 3, time to peak HR after the climb; 4, slowly decreasing HR after the peak; 5, fast decreasing HR.

The HR raw data were synchronized to the stopwatch data, and the following parameters were determined:

(a) Climbing time from start to finish (unit: s); time data of unsuccessful climbs (slipping off the wall) were discarded;
(b) Climbing speed, i.e., climbing time per unit climbing height (unit: m/s);
(c) Peak HR (unit: bpm) related to each climb;
(d) Time to peak HR after each climb (unit: s);
(e) Rate of change in heart rate, i.e., the increase or decrease in the HR per unit time (unit: bpm/s), specifically average bpm/s for the ascent (phase c and 3, Figure 2) and descent (phase 5, Figure 2), and peak bpm/s for ascent;

(f) Increase in HR during the climb and after the climb until peak HR is reached;
(g) average HR (bpm) during the pauses, before the first climb, and after the last climb.

### Statistics

From the data of the different parameters, the averages, standard deviation, minima, and maxima were determined for each of the six climbs across the seven participants, each climbing height, each pause, and each participant.

The averages were compared and significant differences detected (*p* < 0.05), by means of the following statistical tests:

(a) For comparison of averages of two correlated samples (complete datasets only), the Wilcoxon rank-sum test was used, as data specific to each climber had to be compared.
(b) For comparison of averages of two samples (incomplete datasets only), the Mann–Whitney *U* test was used, if data were missing (malfunction of the HR monitor; or unsuccessful climbs), which prevented the comparison of climber-specific data.
(c) For comparison of averages of more than two samples, such as the peak HR (six climbs) and the pause HR (seven pauses in total), the Friedman rank-sum test for multiple correlated samples (complete datasets) was used, followed by the Conover *post hoc* test, with *p*-values adjusted by the Holm familywise error rates (FWERs) and Benjamini–Hochberg false discovery rate (FDR) methods. A test for the correlated sample was selected, as data specific to each climber had to be compared.

## Results

### Heart Rate Profiles

**FIGURE 3:**
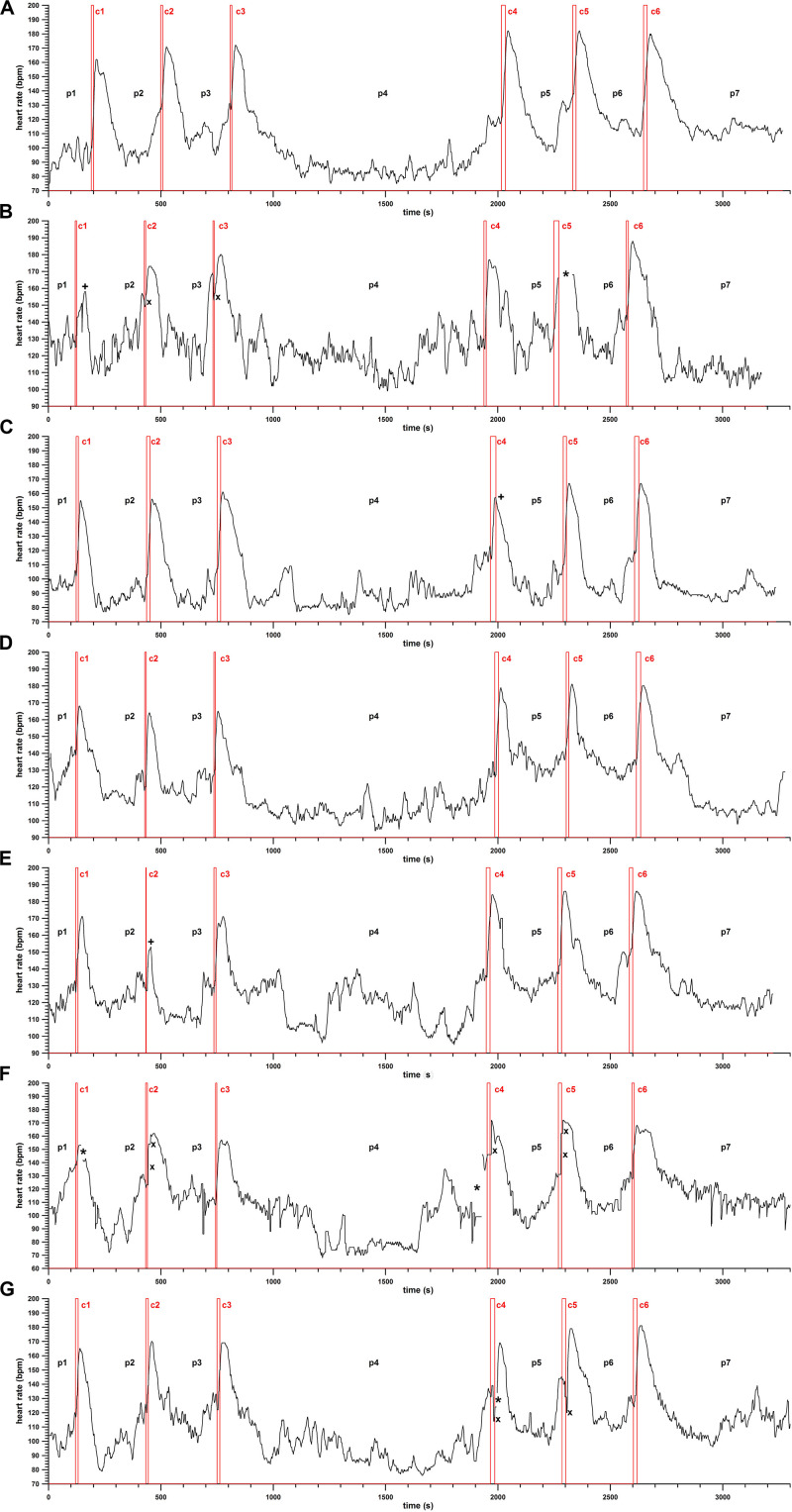
Heart rate (HR; bpm) against time (s), HR profiles of climbers 1–7 (from top to bottom, **A–G**); c1–c6, the six climbs per participant (c1–c3 = 10 m, c4–c46 = 15 m); p1–p7, pauses between the climbs; *, lost data; ×, artifacts; +, unsuccessful climbs.

The HR profiles are shown in Figures 2, 3.

In general, the HR during the pauses between the climbs is in most cases at the steady state (phase 1, Figure 2). Before the climb, the HR increases due to prestart activation immediately before the climb (phase 2, Figure 2). The prestart activation can be missing, as seen in the first and last climb in Figure 3A. The HR starts to increase at the beginning of the climb (c) and continues to rise after the climb until reaching a peak (phase 3, Figure 2). Subsequently, the HR decreases, in most cases with a flatter drop first (phase 4), followed by a steeper one (phase 5).

Figure 3 displays the HR profiles of all participants. Due to rapid and high-intensity movements of the arms and the shoulders, the HR monitor got detached from the skin, either resulting in intermittent data loss or HR artifacts (sudden jumps over tens of beats per minute).

### Climbing Time and Speed

The climbing times of the 10- and 15-m training runs were the following:

10 m: 9.16 ± 3.06 s (5–16 s), average climbing time of 9.16 s over 10 m corresponds to 1.092 m/s.
15 m: 14.95 ± 3.14 s (9–19 s), average climbing time of 14.95 over 15 m corresponds to 1.004 m/s.

Climbing speed of the 10- and 15-m training runs:

10 m: 1.209 ± 0.395 m/s (0.625–1.667 m/s);
15 m: 1.055 ± 0.264 m/s (0.789–1.500 m/s).

Although the average speed over 10 m seems faster, there was no significant difference between the two speed averages (*p* = 0.1096, Mann–Whitney test, *U* = 173.5). The reason why the two speed averages are different from the speed calculated from the two climbing time averages lies in the fact that the climbing speed is a reciprocal function of the climbing time and therefore non-linearly related to the climbing time.

### Peak Heart Rate

The peak heart rate occurs only after the climbing ascent was completed. The statistical data of the peak heart rate are the following:

10 m: 164.57 ± 7.45 bpm (153–180 bpm);
15 m: 176.43 ± 8.09 bpm (157–188 bpm).

The difference between the two averages of the peak heart rate was highly significant (*p* = 0.0001; Wilcoxon signed-rank test, *W* = −225, *n*_s/__r_ = 21, z = −3.9).

**FIGURE 4:**
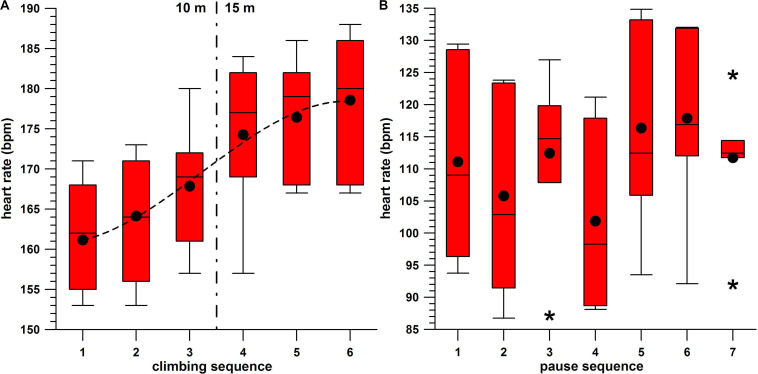
**(A)** Peak heart rate against the sequence of the six climbs per participant, box-and-whisker plots, average (•), and cubic fit function (dashed line); **(B)** pause heart rate against the sequence of the seven pauses per participant, box-and-whisker plots, average (•), and outliers (*).

The individual averages (HR^avg^) at each single consecutive climb seem to increase continuously: 10 m (10a,b,c): HR^avg^_10__a_ = 161.17 bpm, HR^avg^_10__b_ = 164.14 bpm, HR^avg^_10__c_ = 167.86 bpm; 15 m (15a,b,c): HR^avg^_15__a_ = 174.29, HR^avg^_15__b_ = 176.43 bpm, and HR^avg^_15__c_ = 178.57 bpm (Figure 4A).

In order to investigate this further, the Friedman rank sum test for the six correlated HR samples (10a,b,c and 15a,b,c) was significant (*p* = 0.0004), with *post hoc* tests delivering the following significantly (*p* < 0.05) different pairs (“<” and “>” denote a significant difference between two HR^avg^; “=” denotes an insignificant difference): HR^avg^_10__a_ < HR^avg^_15__abc_; HR^avg^_10__b_ < HR^avg^_15__abc_; HR^avg^_10__c_ < HR^avg^_15__bc_; HR^avg^_10__a_ < HR^avg^_10__c_ (FDR method only). HR^avg^_10__c_ = HR^avg^_15__a_, as these two climbs were consecutive ones, and also separated by the 20-min pause.

### Time to Peak Heart Rate After Each Climbing Ascent

The time lag between the completion of the climb and the peak of the heart rate were as follows:

10 m: 12.95 ± 7.10 s (5−31 s);
15 m: 13.32 ± 4.04 s (8–22 s).

There was no significant difference between the two speed averages (*p* = 0.3371; Mann–Whitney test, *U* = 224.5).

### Rate of Change in Heart Rate

The average HR “accelerations” (before reaching the peak HR) were as follows:

10 m: 2.44 ± 0.84 bpm/s (0.99–3.76 bpm/s);
15 m: 2.60 ± 0.77 bpm/s (1.67–4.22 bpm/s).

There was no significant difference between the two average rates of change (*p* = 0.6241; Mann–Whitney test, *U* = 220).

The peak HR “accelerations” (before reaching the peak HR) were as follows:

10 m: 4.24 ± 0.88 bpm/s (2.83–6.33 bpm/s);
15 m: 4.08 ± 1.26 bpm/s (2.33–6.67 bpm/s).

There was no significant difference between the two average rates of change (*p* = 0.5687; Mann–Whitney test, *U* = 291).

The average HR “decelerations” (after the peak HR) were as follows:

10 m: −0.99 ± 0.27 bpm/s (−1.51–0.54 bpm/s);
15 m: −0.96 ± 0.33 bpm/s (−1.66–0.49 bpm/s).

There was no significant difference between the two average rates of change (*p* = 0.4473; Mann–Whitney test, *U* = 209).

### Increase in HR During and After Climb Before the Peak HR

HR increase during the climb:

10 m: 18.33 ± 13.20 bpm (−7–45);
15 m: 29.75 ± 9.38 bpm (10–41).

The difference between the two averages was significant (Mann–Whitney test, *p* = 0.0093, *U* = 220).

HR increase after the climb up to the peak HR:

10 m: 21.82 ± 9.96 bpm (0-37);
15 m: 18.36 ± 7.41 bpm (8–34).

The difference between the two averages was not significant (Mann–Whitney test, *p* = 0.1096, *U* = 97).

Comparing the HR increase during and after the climb:

10 m: not significant (Wilcoxon rank-sum test *p* = 0.2187, *W* = −57), as averages (18.33 and 21.82 bpm) are too close and therefore statistically similar;
15 m: significant (Wilcoxon rank-sum test *p* = 0.0135, *W* = 96), as averages (29.75 and 18.36 bpm) were different.

The reasons for these results are the following:

(1) If neither the average HR “deceleration” nor the average time to peak HR are not different on 10- and 15-m walls, then the HR increase after the climb is not expected to be different either;
(2) The climbing time on the 10-m wall is too short to have the HR increase during the climb exceed the HR increase after the climb.

### Pause Heart Rate

The heart rate data of the seven pauses (Figure 4B) are as follows:

p1: 111.12 ± 14.99 bpm (93.77–129.42);
p2: 105.80 ± 15.84 bpm (86.76–123.82);
p3: 112.43 ± 12.56 bpm (87.19–126.98);
p4: 101.87 ± 13.83 bpm (88.11–121.16);
p5: 116.36 ± 16.36 bpm (93.51–134.83);
p6: 117.89 ± 13.90 bpm (92.13–132.02);
p7: 111.73 ± 9.74 bpm (92.02–124.66).

Comparing the HR of the seven different pauses (p1–p7) with the Friedman rank sum test for seven correlated samples indicated significant differences (*p* = 0.0028).

The *post hoc* tests resulted in the following significantly (*p* < 0.05) different pairs.

The HR^avg^ of the long pause p4 (HR^avg^_p__4_ = 101.87), which was the lowest of all seven pauses, was different from the HR^avg^ of p1, p3, p5, p6, and p7 (p1, p3, and p7 in the FDR method only), but not from HR^avg^_p__2_. HR^avg^_p__2_ (105.8), the second lowest one, was different from the HR^avg^_p__5_ and HR^avg^_p__6_ (116.36 and 117.89), the two highest ones.

The difference between the two HR^avg^ of p2 + p3 combined (two pauses of 10 m, 109.12 bpm) and p5 + p6 (two pauses of 15 m, 117.12 bpm) was highly significant (*p* = 0.003; Wilcoxon signed-rank test, *W* = −95, *n*_s/__r_ = 14, *z* = −2.97).

## Discussion

This paper describes, for the first time, the HR behavior and profiles during speed climbing. The limitations of this study were already outlined at the beginning of the Section “Methods,” resulting in low numbers of participants and gender inequity. In terms of uneven gender distribution, a hypothetical difference in HR behavior between female and male participants could not be evaluated. However, Panissa et al. (2016) investigated the behavior of the HR during all-out high-intensity cycling and did not find a significant difference between 9 female and 10 male participants. Another limitation of our study was that maximum and baseline values of HR of the participants were not determined in a prestudy. This should be included in the protocol of further similar studies.

The main finding is that the HR increases, after climbing a 10- or 15-m wall, for another 13.13 ± 5.74 s (5–31 s). As the primary energy system for short (5–15 s) “all-out” full-body exertions is the ATP-PCr system, and as EPOC is engaged immediately after the climb, the increase in HR is expected to result entirely from the combined effect of psycho-physiological overload.

Another main finding of this research is the behavior of the HR acceleration and deceleration, which has been paid little attention in the literature.

Fisher et al. (1983) investigated “*the maximal rate of*…*tachycardia development*…*to distinguish accurately between sinus and ventricular tachycardia.*” Sinus tachycardia was induced in test persons who “*rushed up 100 stairs as rapidly as possible*,” and found, during the first second of this exercise, that the rate of change in heart rate was 20 bpm/s on average. In contrast to this, Fisher et al. (1983) encountered a far higher rate of change in heart rate in spontaneous episodes of ventricular tachycardia, namely, 88 bpm/s on average. Twenty beats per minute per second within the first second seems excessively high; however, the test persons’ lifestyle activity ranged from sedentary to limited regular physical activity. It was explained to them before the experiments that there is a necessity for sudden maximal effort, and they were encouraged to ascend two stairs with every step. Rushing up a flight of 100 stairs can be compared to speed climbing in terms of vertical movement, insofar as if the height of a stair is 0.2 m, then 100 stairs represent a height of 20 m. However, there is a difference between the participants of our study and the one of Fisher et al. (1983). Speed climbers have a slower start compared to running up a staircase, and our participants were near-elite speed climbers. In fact, in some climbs, we saw a slight decrease in the HR by a couple of beats per minute before the HR rose rapidly. The rate of change in heart rate (HR acceleration) we found was 2.5 bpm/s (average acceleration across the entire HR increase) and 4.2 bpm/s (average peak acceleration).

Knox (1940) used a tolerance test in medical students who were not in training (“*the subject rises to his feet, steps five times up and down two steps each ten inches high and then sits down again and relaxes*”) to determine the “*acceleration of the heart rate in beats-per-minute per second.*” Knox (1940) obtained an average HR acceleration (from baseline to maximum HR) of 3.0 ± 0.9 bpm/s (1.4–5.3 bpm/s). It is surprising that “*in healthy young men performing a very light exercise*” (Knox, 1940) and without any psychological pressure, the average HR acceleration was as high as 3.0 bpm/s, whereas in near-elite speed climbers over an intense, maximal speed and high-power exercise, the average HR acceleration was only 2.5 bpm/s. The average peak HR of both cohorts was 130 bpm (Knox, 1940) and in speed climbing was 165–175 bpm (10 and 15 m, respectively). It can, therefore, be concluded that although an intense exercise in near-elite athletes elicits higher HR, their HR acceleration is nevertheless smaller than in untrained men performing a very light and unstressful exercise. Considering this, the 20 bpm/s found by Fisher et al. (1983) does not seem excessive. It can be hypothesized that the more trained the athletes are and the greater their experiences with accommodating psychological pressure, the slower their HR acceleration is.

Whether or not the 5-min pauses (IFSC Rule 9.14; International Federation of Sports Climbing [IFSC], 2019b) are appropriate between climbs of a speed climbing competition can be addressed in the following way.

Comparing the average HR of the pauses with the Friedman test including the *post hoc* tests delivers the following result. The longer pause (p4) exhibits less HR on average compared to the other six pauses. In addition to this, HR^avg^_p__1_ > HR^avg^_p__4_ (FDR method only), and HR^avg^_p__5_,_6_ = HR^avg^_p__1_. This means that HR^avg^ in the last 3 min before the first climb (and after warming up) is higher than HR^avg^ in the 20-min pause between the 10- and 15-m climbs. However, HR^avg^ of the two pauses p5 and p6 combined (between the three 15-m climbs) is statistically as high as the one of p1. Additionally, from Figure 3A, the HR reaches a steady state (constant signal amplitude, on average) in most of the pauses. From the average data, a 5-min pause seems to be sufficient. This statement is made since the HR after the climbs (in pauses p2–p7) were not significantly different from the average HR before the first climb (p1, which served as a baseline), except for the HR in p4, which was the long pause (20 min) after climb 3.

Addressing this problem from the worst-case scenario (longest possible HR recovery period), instead of from the average data, the following parameters (15-m climb only) have to be considered:

– Time to peak HR: 13.32 ± 4.04 s on average, with a range of 8–22 s;
– Average HR deceleration: −0.96 ± 0.33 bpm/s (−1.66–0.49);
– HR of pauses p5 + p6: 117.12 ± 14.60 bpm (92–135); and
– Peak HR: 176.43 ± 8.09 (157–188).

Out of these parameter ranges, the worst cases (for a prolonged HR recovery period) are 188 bpm peak HR, 22 s time to peak HR after the climb, and the HR drops from 188 to 92 bpm at a rate of −0.5 bpm/s. This drop lasts for 192 s, plus the 22 s time to peak HR results in 214 s or 3.57 min after the climb. This still leaves 1.4 min for a steady-state HR, which does not include the time for prestart activation. In light of this, it can be confidently stated that 5 min recovery time between the climbs is sufficient. This applies to the HR only and not to other physiological parameters. Yet, the limitation of this worst-case scenario is that the data were taken from training climbs. It is unknown whether these data are applicable to the competitions, with a higher level of psychological stress.

## Data Availability Statement

The raw data supporting the conclusions of this article will be made available by the authors to any qualified researcher, if they have obtained Ethics Approval for secondary use of existing data through a Consent Waiver.

## Ethics Statement

The studies involving human participants were reviewed and approved by Swinburne University Human Ethics Committee (approval no. 20191290-1680), Swinburne University, Hawthorn, VIC, Australia. The participants provided their written informed consent to participate in this study.

## Author Contributions

FF, AT, SP, GN, and YW contributed equally to the design of the study, execution of the experiment, performing of data analyses, and writing and editing of the manuscript. All authors contributed to the article and approved the submitted version.

## Conflict of Interest

GN is employed by company Sportstättenverein Marswiese. The remaining authors declare that the research was conducted in the absence of any commercial or financial relationships that could be construed as a potential conflict of interest.
